# Supplementary material for: Spatial transcriptomics reveals metabolic changes underly age-dependent declines in digit regeneration
Source: eLife. 2022 May 26;11:e71542. doi: 10.7554/eLife.71542 (PMC9135401; doi:10.7554/eLife.71542)
Supplement: Transparent reporting form [file elife-71542-transrepform1.pdf]

Westbrook Centre
Milton Road
Cambridge CB4 1YG, UK

P 01223 855340
W elifesciences.org
T @elife

eLife's transparent reporting form

We encourage authors to provide detailed information *within their submission* to facilitate the interpretation and replication of experiments. Authors can upload supporting documentation to indicate the use of appropriate reporting guidelines for health-related research (see EQUATOR Network), life science research (see the BioSharing Information Resource), or the ARRIVE guidelines for reporting work involving animal research. Where applicable, authors should refer to any relevant reporting standards documents in this form.

If you have any questions, please consult our Journal Policies and/or contact us: editorial@elifesciences.org.

Sample-size estimation

- You should state whether an appropriate sample size was computed when the study was being designed
- You should state the statistical method of sample size computation and any required assumptions
- If no explicit power analysis was used, you should describe how you decided what sample (replicate) size (number) to use

Please outline where this information can be found within the submission (e.g., sections or figure legends), or explain why this information doesn't apply to your submission:

*Statistical methods for each analysis can be found in the methods section for each type of assay. Sample size noted in both the methods, the figure legend, and the manuscript text. Sample size is also based on prior publications (see references).

microCT analysis: G*Power Software was used to estimate sample sizes (type I error <5% and power of analysis >80%) to detect an effect size of 0.64 μm in TbSp, which had the greatest variability and lowest explained variance. We require 16 digits, derived from 4 mice per group to detect age differences using ex vivo microCT scanning.
Seahorse: Our current studies utilize 2 mice per plate with experiments in triplicate and assume an 80% power calculation and a significance level of 0.05. With this sample size we have been able to detect changes in OCR (pmol/min/μg protein) as small as 1.3.
Histology and immunohistochemistry images and ghost images (Figure 5H): N=3 individual mice (biological replicates) with representative sample shown. For quantitative IHC data a single section from each slide from each mouse was analyzed. For representative samples the four center sections from each slide from each mouse was evaluated to select the most representative sample.

Replicates

- You should report how often each experiment was performed
- You should include a definition of biological versus technical replication
- The data obtained should be provided and sufficient information should be provided to indicate the number of independent biological and/or technical replicates
- If you encountered any outliers, you should describe how these were handled
- Criteria for exclusion/inclusion of data should be clearly stated

eLife Sciences Publications, Ltd is a limited liability non-profit non-stock corporation incorporated in the State of Delaware, USA, with company number 5030732, and is registered in the UK with company number FC030576 and branch number BR015634 at the address Westbrook Centre, Milton Road, Cambridge CB4 1YG.

- High-throughput sequence data should be uploaded before submission, with a private link for reviewers provided (these are available from both GEO and ArrayExpress)

Please outline where this information can be found within the submission (e.g., sections or figure legends), or explain why this information doesn't apply to your submission:

*Replicates for each analysis can be found in the methods section for each type of assay. Replicates are noted in the figure legend. We utilized the ROUT method to detect and remove outliers (Q=1%).

Seahorse: We evaluated a minimum of three separate Seahorse plates for each analysis, each plate will represent cells from at least 2 separate mice (biological replicates). Technical replicates are individual assay wells indicated in figure legend and methods. Three separate Seahorse plates were assayed in order to confirm metabolic response of cells. Wells that failed to reach confluency at 10 days were removed. BCA protein assay was conducted twice on each well (two separate plates) and then averaged for a single number.
microCT analysis: We utilized 4-7 mice (biological replicates) per time point, per age group. We assayed regenerated digits per mouse, removing digits if they were broken or statistical outliers. OAA treated mice represented 4-7 mice per group (4 digits per mouse).
Histology and immunohistochemistry images and ghost images (Figure 5H): N=3 individual mice with representative sample shown. For quantitative analysis a single section from each slide from each mouse was evaluated.

eLife Sciences Publications, Ltd is a limited liability non-profit non-stock corporation incorporated in the State of Delaware, USA, with company number 5030732, and is registered in the UK with company number FC030576 and branch number BR015634 at the address Westbrook Centre, Milton Road, Cambridge CB4 1YG.

Statistical reporting

- Statistical analysis methods should be described and justified
- Raw data should be presented in figures whenever informative to do so (typically when N per group is less than 10)
- For each experiment, you should identify the statistical tests used, exact values of N, definitions of center, methods of multiple test correction, and dispersion and precision measures (e.g., mean, median, SD, SEM, confidence intervals; and, for the major substantive results, a measure of effect size (e.g., Pearson's r, Cohen's d)
- Report exact p-values wherever possible alongside the summary statistics and 95% confidence intervals. These should be reported for all key questions and not only when the p-value is less than 0.05.

Please outline where this information can be found within the submission (e.g., sections or figure legends), or explain why this information doesn't apply to your submission:

*Statistical methods for each assay can be found under statistical analysis in the Methods section. Statistical test is noted in the methods. N, SD/SEM, p-value and additional information are located in the figure legend or with the figure and in the manuscript text. Graphical representation of Seahorse is presented in standard Seahorse analysis format. Graph format was selected for clarity of data presentation (e.g. line graph over time of microCT does not include individual raw value points).

(For large datasets, or papers with a very large number of statistical tests, you may upload a single table file with tests, Ns, etc., with reference to sections in the manuscript.)

Group allocation

- Indicate how samples were allocated into experimental groups (in the case of clinical studies, please specify allocation to treatment method); if randomization was used, please also state if restricted randomization was applied
- Indicate if masking was used during group allocation, data collection and/or data analysis

Please outline where this information can be found within the submission (e.g., sections or figure legends), or explain why this information doesn't apply to your submission:

*Group allocation approach can be found in the methods section.

microCT: Mice were randomly allocated to either control or treatment groups with equal representation of both sexes. Mice were assigned ear tag numbers and reconstruction, rotation, and analysis final numbers were blinded while being generated.
Seahorse: Mice of equal size were randomly allocated for the assay with equal representation of both sexes and equal distribution to both treatments.
Histology and immunohistochemistry images and ghost images (Figure 4F): group assignment is designated prior to the assay.

Additional data files ("source data")

- We encourage you to upload relevant additional data files, such as numerical data that are represented as a graph in a figure, or as a summary table
- Where provided, these should be in the most useful format, and they can be uploaded as "Source data" files linked to a main figure or table
- Include model definition files including the full list of parameters used
- Include code used for data analysis (e.g., R, MatLab)

eLife Sciences Publications, Ltd is a limited liability non-profit non-stock corporation incorporated in the State of Delaware, USA, with company number 5030732, and is registered in the UK with company number FC030576 and branch number BR015634 at the address Westbrook Centre, Milton Road, Cambridge CB4 1YG.

- Avoid stating that data files are “available upon request”

Please indicate the figures or tables for which source data files have been provided:

Raw and processed files will be made available for public download upon publishing of this manuscript from the Gene Expression Omnibus. All analyses used the standard R package Seurat without the use of newly derived code. SpatialTime analysis was described in detail in the methods section and was conducted in excel on data exported from R.

eLife Sciences Publications, Ltd is a limited liability non-profit non-stock corporation incorporated in the State of Delaware, USA, with company number 5030732, and is registered in the UK with company number FC030576 and branch number BR015634 at the address Westbrook Centre, Milton Road, Cambridge CB4 1YG.
